# Electronic cigarettes use in COVID-19 era among students of a tertiary institution in Nigeria

**DOI:** 10.4102/phcfm.v15i1.3704

**Published:** 2023-01-17

**Authors:** Oluwafunmike A. Ogwa, Abdulhakeem O. Abiola, Oluchi J. Kanma-Okafor, Tolulope F. Olufunlayo, Azeezat O. Shopeyin-Dosunmu, Babatunde A. Akodu, Omonigho D. Ogwa

**Affiliations:** 1 Department of Community Health and Primary Care, Faculty of Clinical Sciences, University of Lagos, Lagos, Nigeria; 2 Department of Medical Services, Doctorkk Health International Ltd, Lagos, Nigeria; 3 Department of Community Health, Lagos University Teaching Hospital, University of Lagos, Lagos, Nigeria

**Keywords:** use of electronic cigarettes, knowledge of e-cigarettes, COVID-19, e-cigarettes, Nigeria, gstudents

## Abstract

**Background:**

Electronic cigarette (e-cigarette) use is becoming popular among undergraduate students in Nigeria with a tendency for escalation because of the coronavirus disease 2019 (COVID-19) pandemic.

**Aim:**

The aim of this study was to assess electronic cigarette use in COVID-19 era among undergraduate students of a tertiary institution in Lagos state, Southwestern Nigeria.

**Setting:**

A tertiary institution in Southwestern Nigeria.

**Methods:**

The study design was a descriptive cross-sectional study with a pretested, structured, close- and open-ended self-administered questionnaire used for collection of data on knowledge, attitude and use of e-cigarettes.

**Results:**

Data from 183 respondents were analysed. The mean age of respondents was 24.8 ± 3.38 years, most (63%) of whom were males. The prevalence of ever-use of e-cigarettes was 15.3% of which 60.7% reported increased use of e-cigarettes since the COVID-19 pandemic. Age, education, tribe and religion (*p* < 0.001) were significantly associated with higher odds of use of e-cigarettes.

**Conclusion:**

This study found an increase in the quantity of e-cigarettes used and online purchase of the same by respondents since the COVID-19 pandemic. Paying attention to the rate of use and having control measures in place for online purchase of e-cigarettes by youths in Nigeria may be necessary in the years to come.

**Contribution:**

This study addresses a gap in the availability of knowledge of use of e-cigarettes among a growing population of youths in Nigeria.

## Introduction

In the year 2020, the world experienced a major public health issue after the report of a cluster of cases of atypical pneumonia in Wuhan, China. It was subsequently discovered to be as a result of a contagious viral infection, initially tagged a novel corona virus (2019-nCoV), but thereafter named severe acute respiratory syndrome coronavirus 2 (SARS-CoV-2) by the World Health Organization (WHO).^1^ With the identification of SARS-CoV-2 and the pandemic that ensued came the implementation of ‘cordon sanitaire’ in many countries to contain the spread of the virus.^1^ This resulted in closure of schools including tertiary institutions and other places of public gatherings in Nigeria consequently tilting some young people towards the use of substances including electronic cigarettes (e-cigarettes).^2,3,4^

Worldwide, tobacco smoking is a major cause of preventable deaths.^5^ Electronic cigarettes (e-cigarettes) are a prototype of devices known as the electronic nicotine delivery systems (ENDS), which are devices that do not burn or use tobacco leaves; rather they vaporise a solution (e-solution) after heating it up.^6^ They were invented by Hon Lik a Chinese pharmacist, as a safer and cleaner way to inhale nicotine as a resource for cessation of smoking tobacco and have been used in many countries with success as part of tobacco cessation programmes.^7^ The e-cigarette comprises three main components: a rechargeable lithium battery, vaporising chamber and a cartridge.^7^ The vapour contains reduced number of harmful chemicals such as carcinogens (formaldehyde, toluene, nitrosamines) that users ingest in comparison to traditional, combustion-based forms of smoking.^8,9^ The vapour may contain nicotine, the active ingredients of marijuana, flavoured propylene glycol, flavoured vegetable glycerin and other flavours (bubble gum and milk chocolate cream), which are likely to be attractive to younger teens.^8^ Nicotine is highly addictive. Although its content is reduced in the e-cigarette, exposure to nicotine can result in damage to the developing brain of adolescents and young adults usually until early to mid-20s and increase the risk of future addiction to other psychoactive substances.^8,10^ Electronic non-nicotine devices (ENNDS) on the other hand do not contain nicotine.^11^

The use of any form of tobacco product, as well as e-cigarettes can be further aggravated when youths are idle, such as the scenario presented by the coronavirus disease 2019 (COVID-19) lockdown, resulting in bulk purchase particularly from online stores.^2^ Physical purchases may result in putting oneself at risk of exposure to COVID-19 by having to make additional trips to stores.^2^ On the other hand, there is a tendency for reduced use of e-cigarettes during the pandemic as a result of having to stay at home with parents, reduced availability of the product or for fear of contracting the virus because of weakened lung function that may be posed by using these products.^3^ The aerosol that users inhale and exhale can expose them and bystanders to harmful substances contained in the vapour.

Globally, there are approximately 367 million users of smokeless tobacco.^12^ Recent surveys in the United States of America (USA) and some European countries have shown marked increases in ENDS use among youths. The overall prevalence from the 2012 WHO estimates for the African region was 12% (22% in males, 2% – 3% in females). The estimated prevalence of smoking in sub-Saharan Africa in 2010 was 14% in males and 2% in females.^13^ According to the Nigeria Demographic and Health Survey (NDHS) conducted in 2018, 0.1% – 0.4% of women aged 15–49 years admitted to smoking cigarettes, while even fewer (0.0% – 0.1%) used other forms of tobacco. With almost a third (31%) of its population being youth, ages 10–24 years, Nigeria boasts of one of the largest youth populations in the world.^14^ Nigeria legislation on tobacco allows the retail sale of e-cigarettes with no restriction to its use: advertising, promotion and sponsorship or packaging and labelling of the same.^12^ This further increased the use of e-cigarettes among youths during the COVID-19 lockdown.^14,15,16^ This study set out to identify and provide relevant data on e-cigarette use by adolescents and young adults who constitute 31% of the Nigerian population,^17^ with the aim of assessing the knowledge, attitude and use of e-cigarettes in the COVID-19 era among students of a tertiary institution in Nigeria.

## Research methods and design

### Study design

The study design was a descriptive cross-sectional study on the knowledge, attitude and use of e-cigarettes among undergraduate students of a tertiary institution in Southwestern Nigeria.

### Setting

The institution where the sample was drawn from was established in 1947 as a quick successor to a higher college but attained an autonomous and independent status in 1969. It has nine schools, 46 departments and about 40 academic programmes offered as full time and part time courses. It currently has a population of approximately 20 000 students. Data collection was completed within two weeks in July 2021.

### Study population and sampling strategy

Sample size of 178 participants was determined using the Cochran’s formula. The level of significance was set at 5% and the prevalence based on a previous study was 12%.^5^ However, 183 respondents who were undergraduate students aged 16 years and above participated in the study. A multistage sampling technique was used for selection of respondents. Firstly, seven out of the nine schools/faculties were selected by simple random sampling method using balloting procedure. Then, three departments were selected from each of the seven schools by simple random sampling method using balloting procedure. Proportionate stratified random sampling method was used to allocate the number of male and female students to participate in each department. Simple random sampling method using balloting procedure was then used to select the participants in the various levels in each department. Undergraduate students >16 years of age at the study location who had completed the school registration process were included in the study.

### Data collection tool

Data collection was carried out using a pretested, structured, close- and open-ended self-administered questionnaire. It was validated and prepared from the Youth Tobacco Survey (YTS) 2011 Questionnaire,^18^ and pretested at Lagos State Polytechnic (LASPOTECH), Isolo campus, Lagos with 20 participants. The questionnaire had four sections. Section A comprised the socio-demographic characteristics of respondents. Section B assessed the adequacy of knowledge of respondents about e-cigarettes during the COVID-19 era. It contained an open-ended question to assess the brands of e-cigarettes known by respondents, as well as close-ended questions to which respondents recorded their responses as ‘yes’, ‘no’, or ‘I don’t know’. Section C assessed the attitude of respondents towards e-cigarettes in the COVID-19 era using a total of 10 questions on a 5-point Likert scale. Each question had five options on a rating scale of 1–5 points for: (1) ‘Strongly agree’, (2) ‘Agree’, (3) ‘Indifferent’, (4) ‘Disagree’ or (5) ‘Strongly disagree’ with the most positive attitude scored as 5 points and others as 4 points, 3 points, 2 points and 1 point, respectively. Section D determined those who have ever or never used e-cigarettes. Those who had never used e-cigarettes did not proceed to answer subsequent questions because the questions applied to use of e-cigarettes. Those who had ever used e-cigarettes proceeded to answer open- and close-ended questions to determine the frequency of use, brands(s) commonly used, influence of COVID-19 restrictions on use and from where they purchased e-cigarettes before and since the COVID-19 pandemic.

### Scoring and grading of knowledge and attitude responses

Each correct response to the knowledge questions scored one mark while incorrect response and no response scored zero. The total score for each respondent was calculated, converted to percentage and graded as poor (< 50%) and good (≥ 50%). The 5-point Likert scale was used to score the attitude responses. Responses to positive attitude statements were scored as follows: (1) strongly agree = 5; (2) agree = 4; (3) indifferent = 3; (4) disagree = 2; (5) strongly disagree = 1, while responses to negative attitude statements were scored as: (1) strongly agree = 1; (2) agree = 2; (3) indifferent = 3; (4) disagree = 4 and (5) strongly disagree = 5. The total attitude score for each respondent was calculated, converted to percentage and graded as poor (≤ 60%) and good (> 60%).

### Data analysis

The data obtained from respondents were coded, entered in a computer system in a Microsoft Excel spreadsheet. EPI INFO 7 software was used for data analysis except regression analysis for which IBM SPSS Statistics for Windows, Version 20.0. Armonk, NY: IBM Corp. was used. Descriptive statistics was used to summarise the data. Independent *t*-test was utilised to determine the differences between continuous quantitative variables. Pearson chi-square test and Fisher Exact test (2-tailed) were used, where applicable to show the association and relationship between categorical variables. Regression analysis was conducted to assess the relationship between statistically significant socio-demographic characteristics and knowledge, attitude and use of e-cigarettes. *P*-value ≤ 0.05 was considered statistically significant.^18^

### Ethical considerations

Ethical approval from the Health Research Ethics Committee of the Lagos University Teaching Hospital was sought and gotten with approval number ADM/DCST/HREC/APP/4264. The consent of the rector of institution where research was conducted was obtained as well as verbal and written informed consent from the participants. Anonymity was maintained during data collection and analysis.

## Results

### Sociodemographic characteristics

All the administered 183 questionnaires were retrieved and analysed giving a response rate of 100%. The mean age of the respondents was 24.84 ± 3.38 years with majority 81 (44.26%) of the respondents being within the age range of 21–25 years. Sixty three per cent (115) were males while 37% (68) were females. A large number came from the faculty of art, design and printing 59 (32.24%) with most participants in ND2 78 (42.62%) and belonging to the Yoruba tribe 113 (61.75%). The predominant religion among respondents was Christianity 107 (58.47%) and most respondents 102 (55.74%) resided off-campus. Most of the respondents 91 (49.73%) had smoking friends while only three (1.60%) of them had smoking mothers (Table 1).

**TABLE 1:** Sociodemographic characteristics and factors affecting knowledge of electronic cigarettes among respondents.

Variables	Total			Knowledge of electronic cigarettes						Statistics		
	*n*	%	Mean ± s.d.	Good (*n* = 88)			Poor (*n* = 95)			*t*	*df*	*p*
				Frequency (*n*)	%	Mean ± s.d.	Frequency (*n*)	%	Mean ± s.d.			
**Sex**												
Male	115	63	-	88	76.52	-	27	23.48	-	-	-	< 0.01
Female	68	37	-	0	0.00	-	68	100	-	-	-	
**Level**												
ND1	27	14.75	-	27	100	-	0	0.00	-	-	-	< 0.001
ND2	76	41.53	-	61	80.26	-	15	19.74	-	-	-	
ND3	13	7.10	-	0	0.00	-	13	100	-	-	-	
HND1	13	7.10	-	0	0.00	-	13	100	-	-	-	
HND2	54	29.51	-	0.00	-	-	54	100	-	-	-	
**Tribe**												
Hausa or Fulani	8	4.37	-	4	50.00	-	4	50.00	-	-	-	< 0.001
Igbo	56	30.60	-	28	50.00	-	28	50.00	-	-	-	
Yoruba	113	61.75	-	52	46.02	-	61	53.98	-	-	-	
Others	6	3.28	-	4	66.67	-	2	33.33	-	-	-	
**Religion**												
Christianity	107	58.46	-	57	53.27	-	50	46.73	-	-	-	< 0.001
Islam	68	37.16	-	28	41.18	-	40	58.82	-	-	-	
Traditional	3	1.64	-	2	66.67	-	1	33.33	-	-	-	
Others	5	2.74	-	1	20.00	-	4	80.00	-	-	-	
**Age (years)**												
< 20	18	9.84	24.84 ± 3.38	18	100	21.90 ± 1.72	0	0.00	27.71 ± 2.10	20.38	181	< 0.001
20–24	81	44.26	-	70	86.42	-	11	13.58	-	-	-	
25–29	77	42.08	-	0	0.00	-	77	100	-	-	-	
30–34	6	3.28	-	0	0.00	-	6	100	-	-	-	
35–39	1	0.55	-	0	0.00	-	1	100	-	-	-	

ND1, National Diploma 1; ND2, National Diploma 2; ND3, National Diploma 3; HND1, Higher National Diploma 1; HND2, Higher National Diploma 2; s.d., standard deviation.

### Knowledge of electronic cigarettes

Fifty-three (28.96%) respondents were of the view that e-cigarettes could aid tobacco smoking, 49 (26.78%) respondents were of the view that e-cigarettes were better than tobacco while majority 99 (54.10%) were of the view that they were an alternative to tobacco smoking. Of the respondents, 87 (47.80%) knew that e-cigarettes could be shaped like a flash drive and 109 (59.56%) were of the view that they were harmful. Majority (62.30%) of the respondents were of the view that e-cigarettes were addictive. The opinion that nicotine is the addictive substance in e-cigarette was held by 88 (48.09%) of the respondents with 118 (64.48%) participants who knew that electronic cigarettes could cause lung cancer. A total of 111 respondents (60.66%) thought that e-cigarettes were not healthy while majority 105 (57.38%) of the respondents felt that e-cigarettes were unlikely to increase an individual’s chances of contracting COVID-19. A total of 95 (51.91%) of the respondents had overall poor knowledge of e-cigarettes while 88 (48.09%) of the respondents had overall good knowledge of e-cigarettes. The respondents’ source of information about e-cigarette was mainly online. However, 151 (83.43%) of the respondents learned about e-cigarettes from friends/peers (Table 2).

**TABLE 2:** Respondents’ knowledge of electronic cigarettes.

Variables	*n*	Frequency (%)
**Statements on knowledge of e-cigarettes (*n* = 183)**		
e-cigarettes can aid tobacco smoking	53	28.96
e-cigarettes are better than tobacco	49	26.78
e-cigarettes are an alternative to tobacco	99	54.10
e-cigarettes are battery-powered	87	47.80
e-cigarettes are shaped like flash drive	49	26.78
e-cigarettes are harmful	109	59.56
e-cigarettes are addictive	114	62.30
Nicotine is the addictive substance in e-cigarettes	88	48.09
e-cigarettes can cause lung cancer	118	64.48
e-cigarettes is not healthy	111	60.66
e-cigarettes does not increase chances of being infected with COVID-19	105	57.38
**Knowledge grade**		
Good	88	48.09
Poor	95	51.91
Total	183	100.00
**Sources of information about e-cigarettes (*n* = 183)**		
Online	147	80.77
Radio	33	18.13
Television	89	48.90
Newspaper	49	27.07
Roadside poster	45	24.73
Friends or peers	151	83.43

COVID-19, coronavirus disease 2019; e-cigarettes, electronic cigarettes.

There were statistically significant relationships (*p* < 0.05) between sex, level of education, tribe, religion as well as age and knowledge of e-cigarettes (Table 1).

### Attitudes towards electronic cigarettes

Among the respondents, 82 (44.81%) strongly disagreed that they would use e-cigarettes if offered by one of their best friends, 78 (42.62%) were not likely to use e-cigarettes in any form in the next 12 months. A total of 52 (28.42%) respondents felt that once someone had started to use e-cigarettes, it would be difficult for the person to quit, 35 (19.13%) respondents strongly disagreed with the opinion that using e-cigarettes made people more comfortable at celebrations, parties and other social gatherings. A total of 67 (36.61%) respondents strongly disagreed that they might enjoy using e-cigarettes, 19 (10.38%) strongly agreed that e-cigarette was an alternative to tobacco smoking and nine (4.92%) strongly agreed that e-cigarettes could help to quit tobacco smoking. Fifty-two (28.42%) of the respondents strongly disagreed that e-cigarettes helped to overcome boredom during the COVID-19 lockdown and 31 (16.94%) respondents thought that COVID-19 pandemic increased the accessibility of e-cigarettes. Thirty-one (16.94%) of the respondents had overall poor attitude towards e-cigarettes, while 152 (83.06%) of the respondents had overall good attitude towards e-cigarettes (Table 3).

**TABLE 3:** Respondents’ attitudes towards electronic cigarettes.

Variables	Frequency (%)									
	SA		A		I		D		SD	
	*n*	%	*n*	%	*n*	%	*n*	%	*n*	%
**Attitude statements (*n* = 183)**										
If one of your best friends offered you e-cigarettes, you would use it	16	8.74	14	7.65	30	16.39	41	22.40	82†	44.81
At any time during the next 12 months, you will likely use a form of e-cigarette	12	6.56	11	6.01	36	19.67	46	25.14	78†	42.62
Once someone has started to use e-cigarettes, it would be difficult for them to quit	52†	28.42	47	25.68	44	24.04	25	13.66	15	8.20
Using e-cigarettes helps people to feel more comfortable at celebrations, parties or in other social gatherings	12	6.56	23	12.57	68	37.16	45	24.59	35†	19.13
I think I might enjoy using e-cigarettes	17	9.29	14	7.65	38	20.77	47	25.68	67†	36.61
E-cigarette is an alternative to tobacco smoking	19†	10.38	32	17.49	68	37.16	43	23.50	21	11.48
E-cigarettes could help to quit tobacco smoking	9†	4.92	12	6.56	80	43.72	51	27.87	31	16.94
E-cigarettes helped to overcome boredom during COVID-19 lockdown	17	9.29	18	9.84	42	22.95	54	29.51	52†	28.42
COVID-19 pandemic made e-cigarettes more available	31†	16.94	50	27.32	42	22.95	27	14.75	33	18.03
**Attitude grade**	-	-	-	-	-	-	-	-	-	-
Good	-	-	-	-	152	83.06	-	-	-	-
Poor	-	-	-	-	31	16.94	-	-	-	-

**Total**	**-**	**-**	**-**	**-**	**183**	**100.00**	**-**	**-**	**-**	**-**

Note: There were statistically significant relationships (*p* < 0.05) between sex, level of education, tribe, religion as well as age and attitude towards e-cigarettes (Table 4).

SA, Strongly agree; A, Agree; I, Indifferent; D, Disagree; SD, Strongly disagree; COVID-19, coronavirus disease 2019; e-cigarette, electronic cigarettes.

† , Most positive attitude.

**TABLE 4:** Factors affecting attitudes towards electronic cigarettes.

Variables	Total			Attitude towards electronic cigarettes						Statistics		
	Frequency (*n*)	%	Mean ± s.d.	Poor (*n* = 31)			Good (*n* = 152)			*t*	*df*	*p*
				Frequency (*n*)	%	Mean ± s.d.	Frequency (*n*)	%	Mean ± s.d.			
**Sex**	-	-	-	-	-	-	-	-	-	-	-	< 0.01
Male	115	62.84	-	31	26.96	-	84	73.04	-	-	-	-
Female	68	37.16	-	0	0.00	-	68	100	-	-	-	-
**Level**	-	-	-	-	-	-	-	-	-	-	-	< 0.001
ND1	27	14.75	-	27	100	-	0	0.00	-	-	-	-
ND2	76	41.53	-	4	5.26	-	72	94.74	-	-	-	-
ND3	13	7.10	-	0	0.00	-	13	100	-	-	-	-
HND1	13	7.10	-	0	0.00	-	13	100	-	-	-	-
HND2	54	29.51	-	0.00	-	-	54	100	-	-	-	-
**Tribe**	-	-	-	-	-	-	-	-	-	-	-	< 0.001
Hausa or Fulani	8	4.37	-	0	00.00	-	8	100	-	-	-	-
Igbo	56	30.60	-	11	19.64	-	45	80.36	-	-	-	-
Yoruba	113	61.75	-	18	15.93	-	95	84.07	-	-	-	-
Others	6	3.28	-	2	33.33	-	4	66.67	-	-	-	-
**Religion**	-	-	-	-	-	-	-	-	-	-	-	< 0.001
Christianity	107	58.46	-	23	21.49	-	84	78.51	-	-	-	-
Islam	68	37.16	-	7	10.29	-	61	89.71	-	-	-	-
Traditional	3	1.64	-	1	33.33	-	2	66.67	-	-	-	-
Others	5	2.74	-	0	0.00	-	5	100	-	-	-	-
**Age (years)**	-	-	24.84 ± 3.38	-	-	20.00 ± 1.24	-	-	25.92 ± 2.90	-	-	< 0.001
16–20	18	9.84	-	2	11.11	-	16	88.89	-	11.14	181	-
21–25	81	44.26	-	81	100	-	0	0.00	-	-	-	-
26–30	77	42.08	-	70	90.91	-	7	9.09	-	-	-	-
31–35	6	3.28	-	0	0.00	-	6	100	-	-	-	-
36–40	1	0.55	-	0	0.00	-	1	100	-	-	-	-

ND1, National Diploma 1; ND2, National Diploma 2; ND3, National Diploma 3; HND1, Higher National Diploma 1; HND2, Higher National Diploma 2; s.d., standard deviation.

### Use of electronic cigarettes

Only 28 (15.30%) of the respondents had ever used e-cigarettes. Among the ever used, 20 (72.41%) were within the age range of 20–39 years. Eleven (39.28%) of the respondents were current users. Most of the respondents who used e-cigarettes 16 (57.14%) used e-cigarettes 1–5 times daily while 20 (74.07%) used e-cigarettes 0–9 times weekly. Majority (82.14%) of the users had used e-cigarettes for 0–29 days within the past 30 days. Most (82.14%) of the respondents used e-cigarettes before the COVID-19 pandemic, while 21 (75%) used e-cigarettes during the lockdown. An increase in the quantity of e-cigarettes used since the COVID-19 pandemic was noticed among majority 17 (60.71%) of the users. The purchase of e-cigarettes was done online by most 11 (39.29%) users before COVID-19 pandemic and 10 (35.71%) of the respondents usually purchased e-cigarettes online since the COVID-19 pandemic (Table 5).

**TABLE 5:** Respondents’ usage of electronic cigarettes.

Use of electronic cigarettes (*n* = 183)	*n*	Frequency (%)
Respondents who ever used electronic cigarettes	28	15.30
**Age at first use**		
10–19	8	27.59
20–39	20	72.41
Currently using e-cigarettes	11	39.28
**Daily use of e-cigarettes**		
1–5 times	16	57.14
Occasionally	4	14.29
Not any more	8	28.57
**Weekly usage of e-cigarettes**		
0–9 times	20	74.07
10–29 times	7	25.93
**Number of days of e-cigarette use in the past 30 days**		
0–29	23	82.14
30–59	5	17.86
Used e-cigarette before the COVID-19 pandemic	23	82.14
Used e-cigarette during the COVID-19 lockdown	21	75.00
Increase in quantity of e-cigarette usage since COVID-19 pandemic	17	60.71
**E-cigarette purchase location before the COVID-19 pandemic**		
Physical stores	8	28.57
Online	11	39.29
Others	9	32.14
**E-cigarette purchase location since the COVID-19 pandemic**		
Physical stores	9	32.14
Online stores	10	35.71
Others	9	32.14

Note: Mean ± s.d = 22.78 ± 3.97.

s.d., standard deviation; e-cigarettes, electronic cigarettes; COVID-19, coronavirus disease 2019.

There were statistically significant relationships (*p* < 0.05) between sex, level of education, tribe, religion as well as age and ever use of e−cigarettes (Table 6).

**TABLE 6:** Factors affecting ever use of electronic cigarettes among respondents.

Variables	Total			Ever used electronic cigarettes						Statistics		
	Frequency (*n*)	%	Mean ± s.d.	Yes (*n* = 28)			No (*n* = 155)			*t*	*df*	*p*
				Frequency (*n*)	%	Mean ± s.d.	Frequency (*n*)	%	Mean ± s.d.			
**Sex**	-	-	-	-	-	-	-	-	-	-	-	< 0.01
Male	115	62.84	-	28	24.35	-	87	75.65	-	-	-	-
Female	68	37.16	-	0	0.00	-	68	100	-	-	-	-
Level	-	-	-	-	-	-	-	-	-	-	-	< 0.001
ND1	27	14.75	-	27	100	-	0	0.00	-	-	-	-
ND2	76	41.53	-	1	1.32	-	75	98.68	-	-	-	-
ND3	13	7.10	-	0	0.00	-	13	100	-	-	-	-
HND1	13	7.10	-	0	0.00	-	13	100	-	-	-	-
HND2	5	29.51	-	0	0.00		54	100	-	-	-	-
**Tribe**	-	-	-	-	-	-	-	-	-	-	-	< 0.001
Hausa or Fulani	8	4.37	-	0	0.00	-	8	100	-	-	-	-
Igbo	56	30.60	-	9	16.07	-	47	83.93	-	-	-	-
Yoruba	113	61.75	-	17	15.04	-	96	84.96	-	-	-	-
Others	6	3.28	-	2	33.33		4	66.67	-	-	-	-
Religion	-	-	-	-	-	-	-	-	-	-	-	< 0.001
Christianity	107	58.46	-	21	19.63	-	86	80.37	-	-	-	-
Islam	68	37.16	-	6	8.82	-	62	91.18	-	-	-	-
Traditional	3	1.64	-	1	33.33	-	2	66.67	-	-	-	-
Others	5	2.74	-	0	0.00		5	100	-	-	-	-
**Age (years)**	-	-	24.84 ± 3.38	-	-	19.89 ± 1.26	-	-	25.83 ± 2.95	-	-	< 0.001
16–20	18	9.84		18	100		0	0.00		10.46	181	-
21–25	81	44.26	-	10	86.42	-	71	13.58	-	-	-	-
26–30	77	42.08	-	0	0.00	-	77	100	-	-	-	-
31–35	6	3.28	-	0	0.00	-	6	100	-	-	-	-
36–40	1	0.55	-	0	0.00	-	1	100	-	-	-	-

ND1, National Diploma 1; ND2, National Diploma 2; ND3, National Diploma 3; HND1, Higher National Diploma 1; HND2, Higher National Diploma 2; s.d., standard deviation.

### Determinants of knowledge, attitude and use of electronic cigarettes

Logistic regression analysis showed that age < 20 years (odds ratio [OR]: −0.37; 95% confidence interval [CI]: −0.36, −0.17; *p* < 0.01), male sex (OR: 0.62; 95% CI: 0.49,0.80; *p* < 0.001), National Diploma (ND) 2 education level (OR −0.25; 95% CI: −0.13, −0.03; *p* < 0.001) and Yoruba tribe (OR: 0.31; 95% CI: 0.11, 0.38; *p* < 0.001) significantly influenced the knowledge of e-cigarettes (Table 7). The attitude towards e-cigarettes was significantly influenced by ND2 education level (OR: −0.47; 95% CI: 0.00, −0.06; *p* < 0.001) and religion (OR: −0.43; 95% CI: 0.00, −0.11; *p* < 0.001) of respondents (Table 7). Furthermore, the use of e-cigarettes was significantly influenced by age < 20 years (OR: −0.64; 95% CI: −0.42, −0.19; *p* < 0.001), ND1 education level (OR −0.52; 95% CI: −0.17, −0.08; *p* < 0.001), Yoruba tribe (OR: −0.56; 95% CI: −0.46, −0.20; *p* < 0.001) and Christianity (OR: −0.89; 95% CI: −0.38, 0.59; *p* < 0.001) (Table 7).

**TABLE 7:** Determinants of knowledge, attitude and ever use of electronic cigarettes among respondents.

Variables	Adjusted odds ratio	95% confidence interval for odds ratio		*p*
		Lower limit	Upper limit	
Knowledge of electronic cigarettes				
Constant	-	−0.669	0.308	0.467
Age	−0.367	−0.357	−0.128	0.000
Sex	0.619	0.487	0.792	0.000
Level	−0.245	−0.131	−0.032	0.001
Tribe	0.308	0.117	0.381	0.000
Religion	−0.040	−0.136	0.076	0.576
**Attitude towards electronic cigarettes**				
Constant	-	1.908	2.302	0.000
Age	−0.182	0.208	−0.232	0.208
Sex	−0.189	0.125	0.041	0.125
Level	−0.467	0.000	−0.056	0.000
Tribe	−0.114	0.403	0.094	0.403
Religion	−0.432	0.000	−0.112	0.000
**Ever used electronic cigarettes**				
Constant	-	1.167	2.131	0.000
Age	−0.637	−0.416	−0.190	0.000
Sex	−0.240	−0.329	−0.028	0.020
Level	−0.522	−0.174	−0.076	0.000
Tribe	−0.562	−0.457	−0.197	0.000
Religion	−0.893	−0.376	0.585	0.000

## Discussion

This study was designed to determine the knowledge, attitude and use of e-cigarettes during the COVID-19 era among undergraduate students of a tertiary institution in Nigeria. The respondents were mainly 21–25 years of age (81%). This followed a similar trend to what was reported in a previous study.^19^ This study had more male participants than females in an approximate ratio of 2:1 probably because more males usually attend colleges of technology in Nigeria than females, because of relative preference for technology-related courses by males. This might also be as a result of an increased interest by males in e-cigarettes than females because males usually constitute more of the smoking population than females. There was a similar sex preponderance in previous studies.^20,21^ Furthermore, the predominant tribe of respondents was Yoruba. This could be attributable to the study being carried out in Lagos State, which is in the Southwestern part of Nigeria owned by and occupied majorly by the Yoruba tribe who are largely Christians. In addition, most of the respondents had friends who smoked.

In this study, the knowledge of e-cigarettes was assessed using open- and close-ended questions. The knowledge of respondents differed on the various aspects of e-cigarette assessed with most people knowing that e-cigarettes can cause lung cancer. Overall, there was a limited knowledge of e-cigarettes among most of respondents. This is contrary to the findings of studies conducted in the USA, Canada, Great Britain, Australia, China and Rwanda, which found a high level of awareness of e-cigarettes up to 90.1%, 76.6%, 54,%, 73%, 88.4% and 74.9%, respectively, among youths in the population.^22,23,24^ Although there is paucity of research on e-cigarettes in Nigeria, the findings of this study was higher than that in a previous study in Nigeria where 40% of respondents demonstrated adequate knowledge of e-cigarettes.^25^ The difference in the level of awareness may be attributed to the limited sample size in the qualitative study carried out in Lagos State. However, exposure to non-tobacco products was found to be higher than that of tobacco products according to a WHO study.^5^ Although studies are emerging to determine the harmful nature of e-cigarettes, many respondents perceived e-cigarettes as being harmful and addictive unlike findings from a study across the European Union where respondents were less likely to perceive e-cigarettes as being harmful.^26^ Most of the respondents gained their knowledge about e-cigarettes From friends and peers and from online sources. This finding was similar to a study in United Kingdom where the respondents obtained their knowledge of e-cigarettes from families, friends and online sources.^27,28^ This can be explained by increased influence of mass media on youths, which was further heightened by the COVID-19 pandemic by keeping people indoors especially during the lockdown, thus increasing the availability.^15,16^ Numerous brands were identified by respondents with Shisha being the brand with the highest frequency contrary to the findings of another study in Nigeria, which found Hookahs as the common brand known because it is considered safe.^25^

The assessment of attitude towards e-cigarettes in this study was assessed using a Likert scale with five responses. Overall, most respondents had a positive attitude towards electronic cigarette with an understanding of its risks. As a result of the increasing popularity of e-cigarettes worldwide, many youths have been found to ignore their potential harmful effects according to a study conducted in the United States of America. Therefore, contrary to the findings of this study, more American youths were found to have a negative attitude towards e-cigarettes by perceiving it as harmless and popular.^29^ Many respondents in this study were unlikely to use e-cigarettes when offered by friends contrary to the favourable disposition exhibited by undergraduate students towards e-cigarettes in a study conducted in Saudi Arabia.^30^ Some respondents in this study reported that they would not enjoy using e-cigarettes, with about 42.62% stating that they were unlikely to use e-cigarettes in the next 12 months. This was not in keeping with the findings in another study in Nigeria where 80% of the participants reported that they would enjoy using e-cigarettes because of the appealing flavours.^25^ This was probably because the recruited participants were current users of e-cigarettes while respondents in this study were mainly non-users of e-cigarettes. Another reason provided by respondents in another study in the USA for a likelihood of enjoying e-cigarettes was because they were ‘trendy’ and a popular product adopted by other youths.^25^ In Nigeria, e-cigarettes have only gained popularity recently hence the attitude towards the product.^31^ E-cigarettes were considered by a few participants as an aid to quitting tobacco smoking. This is similar to findings from two studies in the USA where respondents reported it as an alternative to tobacco smoking.^13,32^

With respect to availability of e-cigarettes before and during COVID-19 era, this study demonstrates that there was increased availability because of the COVID-19 restrictions relative to what obtained before the COVID-19 era. This finding was unlike that of a study conducted in Indonesia which reported a usual availability of e-cigarettes in physical and online stores.^8^ However other studies found increased engagement of youths by mass media platforms during the COVID-19 era because of the stay-at-home regulations resulting in increased availability of e-cigarettes.^15,16^

Few participants reported ever use of e-cigarettes in this study. This is similar to the findings of 15.4% of a Canadian population in a cross-sectional study of those more than 15 years who reported to have ever used e-cigarettes.^28^ On the other hand, only 2.7% of undergraduate students were found in a study in Brazil to have ever used e-cigarettes attributable to the ban of electronic cigarette use in Brazil.^33^ Nigerian legislation on tobacco does not allow the sale of tobacco products online, but permits the retail sale of e-cigarettes with no restriction to its use; advertising, promotion and sponsorship; or packaging and labelling of same.^34^ Most of the respondents in this study who had ever used e-cigarette were 20–39 years of age when they first used it with a mean age 22.78 years ± 3.97. In a study in Canada, ever use of e-cigarettes was reported in those aged 15 years and above.^28^ In this study, 39.28% of respondents who were ever users reported current use while a much less figure was found in an online cross-sectional survey in China where 1.7% of ever users of e-cigarettes were current users.^35^ The brands found in this study to be commonly used by respondents were Shisha and Snoopdoggy. These are smoking items accessible and publicized in an online market in Nigeria as smoking accessories to which young people with interest in digital technology, particularly during the COVID-19 era, had access as the need for physical store visits for procurement was minimized.^36^ However, in another study carried out in Nigeria, the participants used more of Hookah because of their preference for its flavour.^25^ Although most respondents in this study who were e-cigarette users made use of e-cigarettes before the COVID-19 pandemic, 60.71% of respondents reported an increase in their use since the COVID-19 pandemic. In the USA however, there has been a decline in the use of e-cigarettes, according to a national survey, found to be as a result of reduced ability to get e-cigarettes because of the lockdown or as a result of increased awareness of the increased susceptibility to COVID-19 because of impaired functioning of the lungs recently attributed to e-cigarettes. A recent study however found an unclear relationship between e-cigarette use and risk of severe COVID-19.^37^ Some pre-COVID 19 users reported a reduction in their use of e-cigarettes especially during the lockdown in order to prevent their parents from finding out that they used them.^3^ The purchase of e-cigarettes was mainly from online stores as determined by this study, similar to a finding from a study in India where the youth population had access to purchase of e-cigarettes from online stores despite a ban on the product by the Indian government.^38^ Increased access to the internet during the COVID-19 lockdown resulted in increased access to e-cigarettes from an increased online purchase. Physical purchases were considered to result in putting oneself at risk of exposure to COVID-19 by having to make additional trips to stores.^2^

The factors affecting the knowledge, attitude and use of e-cigarettes were assessed based on some selected socio-demographic characteristics which include sex, level, tribe, religion, and age of respondents. Sex was identified in some studies as a factor contributing to knowledge, attitude, and use of e-cigarettes. In this study, more males had good knowledge (100%), better attitude (100%) as well as a higher use (100%) of e-cigarettes. As more males used tobacco and by extension e-cigarettes, they were found to pay more attention to advertisements for e-cigarettes than the female population.^24^ These advertisements are usually present on social media, to which many youths were exposed during the COVID-19 era, particularly during the lockdown that was implemented by many countries to curtail the spread of the disease. E-cigarettes can also be easily bought in stores. This relative availability and increased access with minimal regulation was found to have influenced the initiation of use of e-cigarettes by adolescents in a study conducted with GATS survey data.^24^ The education level of respondents in this study was recorded as National Diploma 1 (ND1), National Diploma 2 (ND2), National Diploma 3 (ND3), Higher National Diploma 1 (HND1) and Higher National Diploma 2 (HND2). The knowledge, attitude and use of e-cigarettes differed across the various levels. The level of education was found to have an impact on the use of tobacco products especially in Nigeria.^11^ Respondents who belonged to the Yoruba tribe had better knowledge, positive attitude, and increased use of e-cigarettes. The religion that most respondents belonged to was Christianity 107 (58.47%), who also had better knowledge, positive attitude, and higher use of e-cigarettes. This was also determined in a study conducted in Nigeria to assess the prevalence and awareness of cigarette smoking among undergraduates where Christianity was found to be the predominant religion of respondents.^11^ The mean age of respondents in this study with adequate knowledge, positive attitude and high use of e-cigarettes was 21.90 years (SD 1.72), 20 years (s.d. 1.24) and 19.89 years (s.d. 1.26), respectively. The knowledge and use of e-cigarettes in another study were found to be higher in older adults aged 18–24 years.^24^ In addition, adolescents and other youths whose family members smoked tobacco cessation products such as e-cigarettes found it easier to use e-cigarettes because of the support they received from relatives.^38^ A statistically significant difference was found in the knowledge, attitude and use of e-cigarettes when compared with the selected socio-demographic characteristics.

A regression analysis of determinants of knowledge of e-cigarettes showed statistically significant association with higher age, male sex, Yoruba tribe and higher educational level. Some studies found an association between age and knowledge of e-cigarettes with older adolescents found to possess a better knowledge of e-cigarettes compared with younger adolescents.^20,39^ A strong association was also found in a study in India with more males having a better knowledge of e-cigarettes than females. A study in China found an association between knowledge of e-cigarettes and level of education with better knowledge among the undergraduates and poor knowledge among the less educated.^27,35,39^ Furthermore, increased awareness about e-cigarettes was found to be more common in males than females according to a South African study.^40^ The age and education level of respondents were found to be significant determinants of their attitude towards e-cigarettes. Adolescents were found to be more likely to receive gifts of e-cigarettes from their peers and their family members as well as being more likely to view advertisements for e-cigarettes because these advertisements are mainly targeted at that age group.^24,31,35^ The regression analysis for the determinants of use of e-cigarettes found a significant influence of age, education level, tribe and religion of respondents.

### Limitations

This study only included students from one tertiary institution hence might not be generalised to the entire student population of other tertiary institutions in Nigeria.

### Recommendation

While the potential health effects of electronic cigarette use on COVID-19 appear unclear, adequate implementation of existing policies is important to prevent possible yet undetermined consequences of its use among the young population who are becoming increasingly aware of the product.

## Conclusion

This descriptive cross-sectional study to assess the knowledge, attitude, and use of e-cigarettes among undergraduate students of a tertiary institution in Nigeria depicted poor knowledge of e-cigarettes, positive attitudes towards e-cigarettes as well as a low usage of e-cigarettes. The determinants of knowledge, attitude and use of e-cigarettes were found to be mainly younger age (< 20 years), male sex, ND education level, Yoruba tribe and Christian adherents. Numerous brands of e-cigarettes exist and are gradually gaining popularity in the Nigerian market.

The COVID-19 pandemic played a role particularly during the lockdown in influencing the knowledge, attitude and use of e-cigarettes among undergraduate students in Nigeria. There was found to be increased quantity of e-cigarettes use during the lockdown along with increased online purchase by the young population who engaged themselves with their mobile devices during the lockdown. A cohort study would however be needed to establish a direct association of this increased use with COVID-19 pandemic. Statistically significant associations were identified in this study between knowledge, attitude and use of e-cigarettes and the socio-demographic characteristics that were assessed in this study.
